# The Impact of Choice of NNRTI on Short-Term Treatment Outcomes among HIV-Infected Patients Prescribed Tenofovir and Lamivudine in Johannesburg, South Africa

**DOI:** 10.1371/journal.pone.0071719

**Published:** 2013-08-07

**Authors:** Kate Shearer, Matthew P. Fox, Mhairi Maskew, Rebecca Berhanu, Lawrence Long, Ian Sanne

**Affiliations:** 1 Health Economics and Epidemiology Research Office, Department of Internal Medicine, School of Clinical Medicine, Faculty of Health Sciences, University of the Witwatersrand, Johannesburg, South Africa; 2 Center for Global Health & Development, Boston University, Boston, Massachusetts, United States of America; 3 Department of Epidemiology, Boston University School of Public Health, Boston University, Boston, Massachusetts, United States of America; 4 Right to Care, Johannesburg, South Africa; 5 Clinical HIV Research Unit, Department of Internal Medicine, School of Clinical Medicine, Faculty of Health Sciences, University of the Witwatersrand, Johannesburg. South Africa; University of Pittsburgh, United States of America

## Abstract

**Introduction:**

Recent WHO guidelines for resource-limited settings recommend tenofovir in first-line antiretroviral therapy (ART) yet there are suggestions that patients receiving nevirapine with tenofovir have worse outcomes than those receiving efavirenz. We sought to compare outcomes among those taking nevirapine vs. efavirenz with tenofovir and lamivudine.

**Methods:**

We analyzed data on ART naïve, non-pregnant patients, ≥18 years old without tuberculosis co-infection, initiating tenofovir with lamivudine and either nevirapine or efavirenz between April 1, 2010 and July 31, 2011 (when South Africa’s public-sector use of tenofovir began) at Themba Lethu Clinic in South Africa. We measured virologic suppression (viral load <400 copies/ml), virologic failure (2 consecutive viral loads >1000 copies/ml), and attrition (death/loss to follow-up) all at 12 months after ART initiation. Modified Poisson regression with robust error estimation was used to estimate risk ratios (RR) and 95% confidence intervals (CI) for predictors of each outcome.

**Results:**

2,254 patients were prescribed efavirenz, 131 nevirapine. Patients were followed a median (range) of 12.0 (0.1–12.0) person-months. 62.2% were female and median (IQR) age was 37.7 years (31.5–44.1). Patients prescribed efavirenz had similar initiating CD4 counts (median 132 for both regimens) but were somewhat more likely to be WHO Stage III or IV (39.6% vs. 33.6%) than those prescribed nevirapine. No difference in attrition was found (aRR: 0.83; 95% CI: 0.49–1.41). Among patients with ≥1 viral load within 1 year on ART, those prescribed nevirapine were as likely to reach virologic suppression (aRR: 0.97; 95% CI: 0.88–1.07) but more likely to experience virologic failure (aRR: 1.84; 95% CI: 1.02–3.31) than those prescribed efavirenz.

**Conclusions:**

Our results support the notion that, among patients prescribed tenofovir and lamivudine, virologic failure is more common among those taking nevirapine than among those taking efavirenz. Longer-term follow up and larger studies will be needed to confirm this finding.

## Introduction

In many resource-limited settings, until recently lamivudine (3TC), stavudine (d4T), and zidovudine (AZT) were the most common nucleoside reverse transcriptase inhibitor (NRTI) choices for first-line antiretroviral treatment (ART). [1], [2] Due to the toxicities associated with AZT and d4T [3]–[5], the World Health Organization’s (WHO) 2010 adult treatment guidelines [6] recommend using tenofovir in first-line therapy. Despite its greater cost, [7] use of tenofovir could increase tolerability and potentially reduce the need for more expensive second-line therapy.

While the change to tenofovir-based regimens continues, it is unclear what the best non-nucleoside reverse transcriptase inhibitor (NNRTI) is to pair with tenofovir. The 2010 WHO treatment guidelines recommended either nevirapine (NVP) or efavirenz (EFV) as they found no evidence of better outcomes with either regimen, but did note the simplicity of the efavirenz regimen (as a once daily pill) and the lower cost of nevirapine. [6] However, there are some suggestions from small studies that patients receiving nevirapine with tenofovir and either lamivudine or emtricitabine are at increased risk of early virologic failure, [8], [9] though the current evidence is weak. [10], [11] If these findings are confirmed, existing guidelines may need to be revised.

In South Africa, the shift to tenofovir-based regimens began in April 2010. [12] While efavirenz is more commonly used with tenofovir, patients can also be prescribed nevirapine. We used data from a large public-sector HIV treatment clinic in South Africa which initiated over 3,000 patients onto ART between April 2010 and July 2011 to assess whether rates of virologic suppression, virologic failure, and attrition over the first year on ART differed between patients taking nevirapine vs. efavirenz along with tenofovir and lamivudine.

## Methods

### Ethics Statement

Approval for analysis of the data was given by the Human Research Ethics Committee (Medical) of the University of the Witwatersrand and by the Institutional Review Board of Boston University. As per the South African Medical Research Council’s Guidelines on Ethics for Medical Research and the Declaration of Helsinki, individual patient consent was not needed. For this retrospective analysis of routine medical records, the analytic datasets were de-identified and no information was included in the dataset that could reveal a patient’s identity.

### Study Site and Population

The data for this analysis come from the Themba Lethu Clinic in Johannesburg, South Africa, [13] which opened in 2004 as a public-sector clinic with NGO support through PEPFAR. Themba Lethu currently follows the 2013 South African National Treatment guidelines. [14] During the period of this analysis, patients were treatment eligible under the 2010 South African National Treatment guidelines with a CD4 count <200 cells/mm^3^ or a WHO Stage IV condition [12].

Under the 2010 guidelines, standard public-sector ART regimens for patients initiating treatment include tenofovir with lamivudine and either nevirapine or efavirenz. Efavirenz is recommended when TB co-infection is present [15] and nevirapine is recommended for women of childbearing age without reliable contraception due to the potential teratogenic effects of efavirenz in the first trimester of pregnancy, despite limited evidence. [16] Women with CD4 counts >200 cells/mm^3^ and men with CD4 counts >400 cells/mm^3^ are cautioned against initiating nevirapine due to increased risk of severe adverse events. [12].

Patient laboratory blood tests (except viral loads) are taken at ART initiation and monitoring tests (viral load, CD4 count) are conducted at six months, twelve months, then yearly thereafter. If a viral load result is between 400 and 1000 copies/ml, a repeat viral load is usually conducted 6 months later. If, however, a viral load result is >1000 copies/ml then a repeat viral load is typically conducted 3 months later. Patient follow-up occurs roughly every other month for the first six months, then every three months if the patient is considered stable. Information on deaths are recorded through passive surveillance and through linkage with the National Vital Registration System (last done in September 2011). [17].

We analyzed data on treatment eligible, ART naïve, non-pregnant patients, ≥18 years old, without tuberculosis co-infection, initiating a regimen containing tenofovir, lamivudine, and either nevirapine or efavirenz between April 1, 2010 and July 31, 2011. Patients with high baseline CD4 counts (>200 cells/mm^3^ for women and >400 cells/mm^3^ for men) initiated on nevirapine were excluded due to increased likelihood of adverse events.

### Study Variables

Our primary exposure was the drug regimen patients were initiated on: tenofovir-lamivudine-nevirapine (TDF-3TC-NVP) or tenofovir-lamivudine-efavirenz (TDF-3TC-EFV). Under treatment guidelines, nevirapine is distributed as a 200 mg tablet taken once daily for two weeks and twice daily thereafter. Efavirenz is prescribed as a once daily 600 mg tablet or as two 200 mg tablets taken once daily for patients weighing less than 40 kilograms. [12].

Our other covariates of interest were age at ART initiation, sex, baseline CD4 count (<50 cells/mm^3^, 50–99 cells/mm^3^, 100–199 cells/mm^3^, ≥200 cells/mm^3^), defined as that CD4 count closest to the date of ART initiation from one year prior to 14 days after the treatment initiation date, WHO Stage (I, II, III, or IV) at ART initiation, defined by the clinician or from analysis of recorded co-infections, and anemia at ART initiation, defined using hemoglobin level (g/dL) as none (> = 13 for men; > = 12 for non-pregnant women), mild (11 to <13 for men; 11 to <12 for non-pregnant women), moderate (8 to <11 for men and non-pregnant women), or severe (<8 for men and non-pregnant women).

Our primary outcomes were virologic suppression (a single viral load <400 copies/ml after at least one month on treatment) and virologic failure (two consecutive viral loads >1000 copies/ml between 2 weeks and 6 months apart at least 4 months after ART initiation). Patients lost to follow up or who died were censored at their last observation. Our secondary outcome was attrition (death or loss to follow-up). Loss to follow up was defined as ≥3 months late for a scheduled clinic visit with no subsequent visit. Our last outcome was regimen switches, defined as a switch of at least one drug from the initiating regimen. Patients were followed for a maximum of twelve months after ART initiation. Person-time accrued from the date of initiation until the treatment outcome was observed. If the treatment outcome was not observed within twelve months of ART initiation, patients were followed until death, transfer, loss to follow up, or completion of one year of follow up.

### Statistical Methods

We analyzed data by baseline treatment regimen. We calculated frequencies of outcomes during the first year on treatment and present these as simple proportions. We also analyzed predictors of treatment outcomes by calculating crude and adjusted risk ratios using modified poisson regression with robust error estimation. [18] We adjusted for sex, age, and univariate predictors of the outcome (p<0.2).

In addition, we conducted two sensitivity analyses. The first examined whether or not the association between nevirapine use and virologic failure remained at a different definition of failure. For this analysis, we defined virologic failure as a single failing viral load >1000 copies/ml after at least 4 months on treatment. The goal of the second sensitivity analysis was to determine if the application of the nevirapine exclusion criteria of high baseline CD4 counts to the efavirenz group would impact the results. For the second analysis, female patients prescribed efavirenz with CD4 counts >200 cells/mm^3^ and male patients prescribed efavirenz with CD4 counts >400 cells/mm^3^ were excluded from the analytical dataset.

## Results

3,390 patients initiated treatment between April 2010 and July 2011. Of those, 2,534 (74.8%) initiated TDF-3TC-EFV while 169 (5.0%) received TDF-3TC-NVP. Patients on nevirapine were more likely to be pregnant than those on efavirenz (8.3% vs. 0.2% among women) and those with TB were more likely to receive efavirenz than nevirapine (10.8% vs. 1.8%). Patients prescribed nevirapine with high baseline CD4 counts (>200 cells/mm^3^ for women and >400 cells/mm^3^ for men), pregnant women, those with TB, and patients who initiated other regimens were removed from further analysis, leaving 2,385 subjects – 2,254 (94.5%) on TDF-3TC-EFV and 131 (5.5%) on TDF-3TC-NVP.

Pregnant women were younger than included patients (median: 31.0 vs. 37.7) while patients co-infected with TB were of a similar age (median: 36.6). Both pregnant women (median: 116; IQR: 73–198) and TB patients (median 70.5; IQR: 24–149) had lower median baseline CD4 counts than included patients (median: 132; IQR: 59–192) and lower baseline hemoglobin levels (pregnant women, median: 10.0; TB patients, median: 9.9; included patients, median: 11.2). While the baseline BMI value for patients co-infected with TB was lower than included patients (median: 19.2 vs. 22.5), pregnant women had a higher median baseline BMI value than included patients (25.3).

Included patients were followed for a total of 2,064 person-years, for a median (range) of 12.0 (0.1–12.0) months per person. Of all included patients, 62.2% were female and the median (IQR) age was 37.7 years (31.5–44.1). Patients prescribed efavirenz had similar initiating CD4 counts (median 132 for both) but were somewhat more likely to be WHO Stage III or IV (39.6% vs. 33.6%) at ART initiation than those prescribed nevirapine (Table 1).

**Table 1:** Patient characteristics at ART initiation and treatment outcomes for patients initiating a tenofovir based regimen at the Themba Lethu Clinic in Johannesburg, South Africa.

Variable	Exposure	TDF-3TC-EFV	TDF-3TC-NVP
Total N		2254 (100%)	131 (100%)
Sex	Male	846 (37.5%)	56 (42.7%)
	Female	1408 (62.5%)	75 (57.3%)
Age	Median (IQR)	37.8 (31.8–44.3)	33.1 (28.1–40.4)
	<30	391 (17.4%)	41 (31.3%)
	30–34	458 (20.3%)	31 (23.7%)
	35–39	476 (21.1%)	26 (19.9%)
	40–44	402 (17.8%)	14 (10.7%)
	≥45	527 (23.4%)	19 (14.5%)
CD4 count (cells/mm^3^)	Median (IQR)	132 (58–194)	132 (66–179)
	Missing	105 (4.6%)	10 (7.6%)
	<50	480 (21.3%)	20 (15.3%)
	50–99	353 (15.7%)	28 (21.4%)
	100–199	844 (37.4%)	62 (47.3%)
	≥200	472 (21.0%)	11 (8.4%)
WHO Stage	Stage I	837 (37.1%)	62 (47.3%)
	Stage II	525 (23.3%)	25 (19.1%)
	Stage III	527 (23.4%)	28 (21.4%)
	Stage IV	365 (16.2%)	16 (12.2%)
BMI	Median (IQR)	22.5 (19.7–25.9)	23.0 (20.2–25.6)
	Missing	299 (13.3%)	24 (18.3%)
	<18.5	273 (12.1%)	10 (7.6%)
	18.5–24.9	1089 (48.3%)	60 (45.8%)
	25–29.9	400 (17.8%)	29 (22.1%)
	≥30	193 (8.6%)	8 (6.1%)
Hemoglobin	Median (IQR)	11.1 (9.7–12.5)	11.5 (10.2–13.4)
Anemia	Missing	242 (10.7%)	17 (13.0%)
	No Anemia	528 (23.4%)	40 (30.5%)
	Mild Anemia	512 (22.7%)	25 (19.1%)
	Moderate Anemia	798 (35.4%)	42 (32.1%)
	Severe Anemia	174 (7.7%)	7 (5.3%)
Outcome at 12 months	Alive	1718 (76.2%)	101 (77.1%)
	Dead	107 (4.8%)	5 (3.8%)
	Lost to follow-up	289 (12.8%)	14 (10.7%)
	Transferred out	140 (6.2%)	11 (8.4%)
Achieved viral suppression*	Yes	1512 (86.5%)	81 (79.4%)
Experienced virologic failure+	Yes	101 (6.1%)	16 (16.2%)

* Among patients with at least 1 viral load between 1 month and 1 year after ART initiation.

+ Among patients with at least 1 viral load after 4 months on treatment.

### Attrition

Among the 2,385 patients, 415 (17.4%) died or were lost to follow up within one year of ART initiation in a median (IQR) of 4.7 (3.9–7.6) months. No difference in overall attrition was found between patients who received nevirapine compared to those who received efavirenz both before (RR: 0.83; 95% CI: 0.54, 1.26) and after adjusting for baseline characteristics (aRR: 0.83; 95% CI: 0.49, 1.41). (Table 2)

**Table 2:** Unadjusted and adjusted predictors of attrition, virologic suppression, and treatment failure among patients initiated on a tenofovir-based ART regimen between April 2010 and June 2011 at the Themba Lethu Clinic in Johannesburg, South Africa.

Attrition within 12 months (n = 2385)				Suppression within 12 months (n = 1850)			Failure within 12 months (n = 1769)		
Characteristic	Attrition/N (%)	Unadjusted RR (95% CI)	Adjusted RR (95% CI)	Suppression/N (%)	Unadjusted RR (95% CI)	Adjusted RR (95% CI)	Failures/N (%)	Unadjusted RR (95% CI)	Adjusted RR (95% CI)
***Regimen***									
TDF-3TC-EFV	396/2254 (17.6%)	**Reference**	**Reference**	1512/1748 (86.5%)	**Reference**	**Reference**	101/1670 (6.1%)	**Reference**	**Reference**
TDF-3TC -NVP	19/131 (14.5%)	0.83 (0.54, 1.26)	0.83 (0.49, 1.41)	81/102 (79.4%)	0.92 (0.83, 1.02)	0.97 (0.88, 1.07)	16/99 (16.2%)	2.67 (1.64, 4.35)	1.84 (1.02, 3.31)
***Sex***									
Male	213/902 (23.6%)	1.73 (1.46, 2.06)	1.64 (1.28, 2.09)	536/644 (83.2%)	0.95 (0.91, 0.99)	0.95 (0.91, 1.00)	45/620 (7.3%)	1.15 ( 0.81, 1.66)	1.19 (0.80, 1.77)
Female	202/1483 (13.6%)	**Reference**	**Reference**	1057/1206 (87.7%)	**Reference**	**Reference**	72/1149 (6.3%)	**Reference**	**Reference**
***Age at initiation***									
<30	88/432 (20.4%)	**Reference**	**Reference**	273/324 (84.3%)	**Reference**	**Reference**	30/309 (9.7%)	**Reference**	**Reference**
30–34	77/489 (15.8%)	0.77 (0.59, 1.02)	0.68 (0.47, 0.97)	321/390 (82.3%)	0.98 (0.91, 1.04)	0.98 (0.92, 1.05)	32/369 (8.7%)	0.89 (0.56, 1.44)	0.98 (0.59, 1.63)
35–39	86/502 (17.1%)	0.84 (0.64, 1.10)	0.72 (0.51, 1.02)	338/392 (86.2%)	1.02 (0.96, 1.09)	1.02 (0.96, 1.09)	24/380 (6.3%)	0.65 (0.39, 1.09)	0.66 (0.38, 1.16)
40–44	77/416 (18.5%)	0.91 (0.69, 1.20)	0.81 (0.57, 1.15)	271/314 (86.3%)	1.02 (0.96, 1.09)	1.04 (0.97, 1.11)	13/299 (4.4%)	0.45 (0.24, 0.84)	0.39 (0.18, 0.83)
≥45	87/546 (15.9%)	0.78 (0.60, 1.03)	0.71 (0.51, 1.00)	390/430 (90.7%)	1.08 (1.02, 1.14)	1.06 (1.00, 1.12)	18/412 (4.4%)	0.45 (0.26, 0.79)	0.61 (0.33, 1.11)
***Baseline CD4+ Count***									
<50	131/500 (26.2%)	2.18 (1.64, 2.89)	1.42 (1.00, 2.02)	257/341 (75.4%)	0.82 (0.77, 0.88)	0.84 (0.78, 0.90)	49/329 (14.9%)	4.70 (2.55, 8.69)	4.77 (2.41, 9.46)
50–99	75/381 (19.7%)	1.64 (1.20, 2.25)	1.43 (0.98, 2.08)	231/283 (81.6%)	0.89 (0.84, 0.95)	0.91 (0.85, 0.97)	23/270 (8.5%)	2.69 (1.36, 5.31)	2.78 (1.32, 5.83)
100–199	112/906 (12.4%)	1.03 (0.76, 1.39)	1.15 (0.82, 1.62)	678/751 (90.3%)	0.99 (0.95, 1.03)	0.99 (0.95, 1.03)	28/731 (3.8%)	1.21 (0.62, 2.35)	1.26 (0.61, 2.61)
≥200	58/483 (12.0%)	**Reference**	**Reference**	374/409 (91.4%)	**Reference**	**Reference**	12/379 (3.2%)	**Reference**	**Reference**
***WHO Stage***									
Stage I	107/899 (11.9%)	**Reference**	**Reference**	636/744 (85.5%)	**Reference**	**Reference**	49/716 (6.8%)	**Reference**	**Reference**
Stage II	69/550 (12.6%)	1.05 (0.79, 1.40)	1.01 (0.74, 1.37)	412/455 (90.6%)	1.06 (1.02, 1.10)	1.06 (1.02, 1.11)	23/429 (5.4%)	0.78 (0.48, 1.27)	--
Stage III	107/555 (19.3%)	1.62 (1.27, 2.07)	1.08 (0.81, 1.45)	354/417 (84.9%)	0.99 (0.94, 1.04)	1.02 (0.97, 1.08)	29/400 (7.3%)	1.06 (0.68, 1.65)	--
Stage IV	132/381 (34.7%)	2.91 (2.32, 3.65)	0.97 (0.67, 1.40)	191/234 (81.6%)	0.95 (0.89, 1.02)	1.01 (0.95, 1.09)	16/224 (7.1%)	1.04 (0.61, 1.80)	--
***Anemia***									
No Anemia	65/568 (11.4%)	**Reference**	**Reference**	425/479 (88.7%)	**Reference**	--	19/462 (4.1%)	**Reference**	**Reference**
Mild Anemia	62/537 (11.6%)	1.01 (0.73, 1.40)	0.94 (0.66, 1.35)	375/440 (85.2%)	0.96 (0.91, 1.01)	--	31/420 (7.4%)	1.79 (1.03, 3.13)	1.51 (0.87, 2.63)
Moderate Anemia	150/840 (17.9%)	1.56 (1.19, 2.05)	1.53 (1.12, 2.09)	558/652 (85.6%)	0.96 (0.92, 1.01)	--	48/622 (7.7%)	1.88 (1.12, 3.15)	1.35 (0.78, 2.33)
Severe Anemia	60/181 (33.2%)	2.90 (2.13, 3.94)	2.09 (1.37 (3.19)	98/116 (84.5%)	0.95 (0.88, 1.04)	--	8/114 (7.0%)	1.71 (0.77, 3.80)	1.17 (0.51, 2.69)
***BMI***									
<18.5	66/283 (23.3%)	1.54 (1.20, 1.98)	1.24 (0.95, 1.63)	166/201 (82.6%)	0.96 (0.90, 1.03)	0.97 (0.91, 1.05)	16/192 (8.3%)	1.17 (0.69, 1.99)	--
18.5–24.9	174/1149 (15.1%)	**Reference**	**Reference**	809/941 (86.0%)	**Reference**	**Reference**	64/902 (7.1%)	**Reference**	--
25–29.9	43/429 (10.0%)	0.66 (0.48, 0.91)	0.82 (0.58, 1.16)	327/365 (89.6%)	1.04 (1.00, 1.09)	1.01 (0.96, 1.06)	18/346 (5.2%)	0.73 (0.44, 1.22)	--
≥30	9/201 (4.5%)	0.30 (0.15, 0.57)	0.43 (0.21, 0.87)	153/174 (87.9%)	1.02 (0.96, 1.09)	0.99 (0.93, 1.05)	7/171 (4.1%)	0.58 (0.27, 1.24)	--

### Viral Suppression

Among the 1,850 patients who had at least one viral load measurement between one month and one year after ART initiation, viral suppression was common. 1,593 (86.1%) achieved viral suppression and, after adjusting for baseline characteristics, those prescribed nevirapine were as likely to achieve suppression as those prescribed efavirenz (aRR: 0.97; 95% CI: 0.88, 1.07). (Table 2)

### Virologic Failure

1,769 patients had ≥1 viral load measurement after four months of follow up. Of those, 1105 (62.5%) had just one viral load measurement while 553 (31.3%) had two viral loads conducted and 111 (6.3%) had 3 or more viral load measurements. Of the 1,769 included patients, 117 (6.6%) experienced virologic failure. In an unadjusted model, patients on nevirapine were almost 3 times more likely to fail than patients prescribed efavirenz (RR: 2.67; 95% CI: 1.64, 4.35). However, after adjusting for baseline characteristics, the effect was less pronounced but those on nevirapine were still 80% more likely to experience virologic failure than those on efavirenz (aRR: 1.84; 95% CI: 1.02, 3.31) (Table 2); though, with limited sample size, our estimates were imprecise and may overestimate the association. When further limited to those 1,514 patients who achieved viral suppression and had four months of follow up, 15 (1.0%) patients failed, all but one of whom were prescribed efavirenz.

### Regimen Switches

Of the 2,385 patients included in the analysis, 241 (10.1%) switched at least one drug in their regimen within one year of ART initiation. 29.0% of nevirapine patients (n = 38) switched compared to 9.0% of efavirenz patients (n = 203). For patients initiated on efavirenz, the most common switch was from tenofovir to stavudine (26.6%), followed by efavirenz to lopinavir/ritonavir (20.2%). For nevirapine patients, the most common switch was from nevirapine to lopinavir/ritonavir (36.8%), followed by nevirapine to efavirenz (26.3%).

### Sensitivity Analyses

Among the 1,769 patients included in the failure analysis, 360 (20.4%) had at least one failing viral load between 4 and 12 months after treatment initiation. More nevirapine patients (28.3%) than efavirenz patients (19.9%) experienced a single failing viral load resulting in an unadjusted risk ratio of 1.42 (95% CI: 1.02, 1.98). After adjusting for baseline characteristics, an increased risk of approximately 40% for nevirapine compared to efavirenz patients remained. (aRR: 1.44; 95% CI: 1.04, 2.01).

Of the 2254 patients initiated on efavirenz who were included in the analytical dataset, 265 (11.8%) people with high baseline CD4 counts (263 women with CD4 >200 cells/mm3 and 2 men with CD4 >400 cells/mm3) were excluded. 1,504 patients were included in the failure analysis who had at least one viral load after 4 months on treatment. Using our original definition requiring two consecutive failing viral loads >1000 copies/ml, 112 (7.4%) patients failed, 96 (6.8%) of whom were initiated on efavirenz and 16 (16.2%) on nevirapine. Both before (RR 2.37; 95% CI: 1.45, 3.85) and after adjustment (aRR: 2.29; 95% CI: 1.40, 3.74) for baseline characteristics, patients initiated onto nevirapine were over twice as likely to fail as efavirenz patients.

## Discussion

Concern over use of nevirapine with tenofovir was raised in 2004 when a small clinical trial noted patients on tenofovir-lamivudine-nevirapine experienced a high rate of failure after 24 weeks on therapy despite high adherence levels. [19] Similar concerns over failure of nevirapine combined with tenofovir were raised in 2008 with results from Lapadula et al. [8] and in 2009 with the DAUFIN study, both of which were terminated early. [9] Both noted high levels of virologic failure at 12 weeks in patients taking both nevirapine and tenofovir; 19.4% in DAUFIN and 42.9% in the Lapadula study. A study in Nigeria, found that 16.1% (n = 131/813) of patients who received tenofovir-lamivudine-nevirapine failed within 12 months of ART initiation compared to 9.8% (n = 41/417) of patients who received tenofovir-lamivudine-efavirenz. [20].

Other studies have reported conflicting results. A small study of tenofovir with either lamivudine or emtricitabine and nevirapine found that at 24 weeks, all patients were virally suppressed at <400 copies/ml. [21] Other studies have noted virologic failure among patients on tenofovir with lamivudine or emtricitabine and nevirapine ranging from 5.7%–15% among cohorts containing both ART-naïve and treatment experienced patients. [22]–[24] A recent review on the topic concluded that while tenofovir-lamivudine-nevirapine will likely be the most commonly used first-line regimen due to cost, if failure is higher with this regimen, overall costs may be greater than using more costly first-line regimens because of the increased need for second-line regimens. [24].

We found low rates of failure (6.6%) in this cohort receiving tenofovir-lamivudine-nevirapine; in keeping with the findings presented in several previous studies. However, our study does support the notion that among patients prescribed tenofovir and lamivudine, virologic failure is more common among those taking nevirapine than among those taking efavirenz. Over twelve months of follow up, we found that while achievement of virologic suppression was comparable between regimens at over 75% for both nevirapine and efavirenz based regimens, patients initiated on nevirapine were almost twice as likely to experience virologic failure as patients initiated on efavirenz. The results of previous studies showed smaller effects than our findings and may suggest our results are overestimates.

Our study has several strengths. We were able to compare nevirapine and efavirenz directly in a single study and were able to reduce confounding by indication by excluding patients who would be prescribed a regimen due to pregnancy or tuberculosis. Still our findings should be considered along with several limitations. First, we only had data on 131 patients who were prescribed nevirapine along with tenofovir and this led to imprecise estimates. There is also some evidence emtricitabine in combination with nevirapine and tenofovir may be comparable to regimens including both tenofovir and efavirenz, [24] however, no patients in our cohort were prescribed emtricitabine with nevirapine and tenofovir so we were unable to examine this hypothesis. Second, despite removing pregnant women and TB patients some residual confounding may have led to overestimates of measures of effect. Additionally, as data on antiretroviral therapy for prevention of mother-to-child transmission is limited in our dataset, we were unable to control for the effect of prior exposure to NVP in our analyses. Third, we lack a measure of adherence in our dataset leaving us unable to control for its effect on virologic failure. Fourth, ascertainment of death through linkage with the national death registry is only available for South African nationals who provide a valid national identification number. As only 61% [17] of patients meet this criteria, we may underestimate mortality in this cohort. Furthermore, as linkage took place in September 2011, deaths occurring after matching may not be captured in our dataset.

## Conclusion

Treatment failure among patients receiving tenofovir-lamivudine-nevirapine has been high in several studies compared to tenofovir-lamivudine-efavirenz, including our own. Combined with the lack of strong evidence of teratogenic effects of efavirenz, the results of these studies have prompted many programs to favor efavirenz. Our results support this decision. However, many patients who have already initiated nevirapine will likely remain on this regimen and continued follow up of these patients is necessary.
